# Addressing viral hepatitis C reinfections in a low-threshold programme for people who inject drugs in Slovenia

**DOI:** 10.1186/s12954-025-01164-5

**Published:** 2025-02-13

**Authors:** Jasna Černoša, Jelka Meglič Volkar, Mario Poljak, Maja Pohar Perme, Jeffrey Victor Lazarus, Mojca Matičič

**Affiliations:** 1 Clinic for Infectious Diseases and Febrile Illnesses, University Medical Centre Ljubljana, Ljubljana, Slovenia; 2 Institute of Microbiology and Immunology, Faculty of Medicine, University of Ljubljana, Ljubljana, Slovenia; 3 Department of Biostatistics and Medical Informatics, Faculty of Medicine, University of Ljubljana, Ljubljana, Slovenia; 4 Barcelona Institute for Global Health (ISGlobal), Hospital Clínic, University of Barcelona, Barcelona, Spain; 5 City University of New York Graduate School of Public Health and Health Policy (CUNY SPH), New York, NY USA; 6 Faculty of Medicine and Health Sciences, University of Barcelona, Barcelona, Spain; 7 Faculty of Medicine, University of Ljubljana, Ljubljana, Slovenia

**Keywords:** Hepatitis C, Micro-elimination, People who inject drugs, Low-threshold settings, Reinfection, Slovenia

## Abstract

**Background:**

Hepatitis C virus (HCV) infection remains a public health threat. Although therapy with direct-acting antivirals made its elimination possible, major challenges remain in treating vulnerable populations, such as people who inject drugs (PWID) enrolled in low-threshold programmes (LTPs). This study analysed the outcome of HCV management focused on HCV reinfection in a specifically designed model-of-care (MoC) for PWID in Slovenia, where treatment is prescribed without limitations, though only by specialist physicians.

**Methods:**

All HCV antibody (anti-HCV) positive users of a MoC, combining HCV management at Clinic for Infectious Diseases at the University Medical Centre in Ljubljana and LTP for PWID in 100 km distanced civil society organisation (CSO) Svit Koper, between January 2017 to December 2022, were included. The MoC enabled regular transportation of PWID between LTP and the Clinic, where specifically assigned services for individually tailored HCV management in cooperation with CSO were available. Data on participants´ demographic, epidemiological, and clinical characteristics were collected partly retrospectively and prospectively, with a particular focus on HCV treatment outcome and reinfection status, and analysed accordingly.

**Results:**

The study included 49 anti-HCV positive PWID with a mean age of 38.7 (standard deviation (SD) = 7.6) years at first visit. The majority was male (40/49, 81.6%); 16/49 (32.7%) experienced previous incarceration, 14/49 (28.6%) were experiencing homelessness, and 42/49 (85.7%) were receiving opioid agonist therapy. A total of 42/49 (83.7%) were HCV RNA-positive. Of them 36/42 (85.7%) started HCV treatment at a mean age of 42.7 (SD = 5.7) years and 33/36 (91.7%) completed treatment. Six (14.3%) HCV RNA-positive PWID died. Among 28/33 (84.9%) who achieved a sustained virological response 12 weeks post treatment, 6/28 (21.4%) presented with reinfection. The HCV reinfection rate was 13.3 per 100 – PY (95% confidence interval (CI) [6.0, 29.7]), the rate of positive HCV RNA re-test was 12.2 per 100 – PY (95%CI [7.7–16.7]), while hazard of reinfection in our cohort increased with time, with the estimated reinfection probability exceeding 0.5 at 4 years.

**Conclusions:**

In marginalised population of PWID attending LTP, a sustainable HCV RNA re-screening and follow-up after HCV cure are necessary, as the risk of reinfection remains high.

**Supplementary Information:**

The online version contains supplementary material available at 10.1186/s12954-025-01164-5.

## Introduction

Hepatitis C virus (HCV) infection is a major public health problem worldwide, as it is a leading cause of chronic hepatitis, cirrhosis and hepatocellular carcinoma, as well as the most common cause for liver transplantation in many high-income countries [1]. The number of chronically HCV infected people is estimated at 50 million globally and most of them are asymptomatic and remain unaware of their infection until severe and irreversible liver disease develops [1].

Therapy with direct-acting antivirals (DAAs) has made HCV elimination and, consequently, the reduction of the burden of chronic liver disease, such as cirrhosis, possible. However, inequity due to their marginalisation impedes HCV elimination, as demonstrated by the fact that in developed countries vulnerable populations, such as people who inject drugs (PWID), experience the highest prevalence of HCV infection. This is a result of improper access to healthcare services due to barriers faced by patients, healthcare practitioners, healthcare systems and health policy-makers, such as limited locations for HCV testing and treatment, lack of financial resources for travel costs, insurance restrictions on HCV therapy coverage for PWID with active substance use, stigmatisation of PWID in healthcare system, and some others [2–5]. Besides, even when receiving HCV treatment and cure, reinfections in this population due to continuous risk exposure were shown to be more common [6–8].

Under the micro-elimination approach, in which national level elimination goals are broken down into smaller elimination targets for individual populations, different models-of-care (MoCs) have been developed to best meet the needs of specific high-risk groups across different settings, taking into account the existing specificities of each country and its policies [3, 9]. In Slovenia, a small high-income central European country with a population of 2.1 million and estimated median HCV RNA prevalence in general population of 0.07%, in accordance with national clinical practice guidelines, HCV therapy is fully covered by the national health insurance system without any restrictions, yet it can be solely prescribed by specialists [10, 11]. With the advent of DAAs, HCV test-and-treat strategies have been introduced, accelerating its elimination nationally. HCV micro-elimination has been achieved in Slovenia among several easily accessible high-risk groups (i.e. people living with haemophilia, haemodialysis patients and people living with an HIV-coinfection) [12].

Among the estimated nearly 5000 PWID residing in Slovenia, over 60% are managed by high-threshold opioid agonist treatment (OAT) programmes operating within a national network of 21 Centres for the Prevention and Treatment of Illicit Drug Use (CPTIDUs). Via CPTIDUs, PWID are offered information, counselling, HCV screening, including confirmatory testing, and, in case of an active HCV infection, referral to a specialist for further assessment and treatment with no restrictions [13, 14]. In 2006, the HCV RNA prevalence across all CPTIDUs was 16%. After the introduction of the national guidelines for HCV management in PWID in 2007, this figure decreased [15], with the latest modelling projections in 2019 estimating an HCV RNA prevalence of 5.2% among former PWID and 13.5% among active PWID [11].

In addition to CPTIDUs, there are also 16 civil society organisations (CSOs) that run low-threshold programmes (LTPs) for PWID [16]. These LTPs focus on harm reduction through fieldwork involving the provision of information and counselling on HCV infection, as well as syringe, needle, and clean drug paraphernalia distribution, without expectations of abstinence from drug use. Currently, in Slovenia PWID managed at CSOs represent a key target group for improvements in HCV screening, linkage-to-care and treatment. As such, in 2017 the referential viral hepatitis centre at the Clinic for Infectious Diseases and Febrile Illnesses at the University Medical Centre in Ljubljana started a collaboration with the CSO Svit Koper from a 100 km distanced Koper and developed a new MoC to improve the HCV continuum-of-care for PWID [17]. The aim of this study was to evaluate the cascade of HCV care for PWID managed within MoC, focusing primarily on the occurrence of HCV reinfections.

## Methods

This was partly a retrospective and partly a prospective study of all HCV antibody (anti-HCV) positive PWID who used the MoC between January 2017 and December 2022. Participants were managed for HCV infection at the Clinic for Infectious Diseases and Febrile Illnesses at the University Medical Centre in Ljubljana. During the first two years of the study, data were conducted retrospectively, which was then followed by prospective data collection. Data on participants’ demographic, epidemiological, clinical, and virological characteristics, as well as their DAA therapy outcomes, were obtained from medical records. Particular attention was given to identifying and analyzing cases of HCV reinfection following sustained virological response 12 weeks after treatment (SVR12). Additionally, available demographic data on all PWID, enrolled in the Svit Koper LTP between 2017 and 2022, were collected retrospectively from the registry of Svit Koper LTP users.

### Setting

Svit Koper is a CSO that operates as the only LTP for PWID in the small coastal region in Slovenia, focusing on reducing the harm caused by drug use via a day centre where PWID can anonymously receive free injecting equipment and have the opportunity to get counselling and practical help with social, medical or other problems through a nurse and social worker. The LTP also runs a mobile unit that offers a needle/syringe exchange programme and operates in coastal municipalities [18]. The closest viral hepatitis treatment centre is located at the Clinic for Infectious Diseases in Ljubljana, which is a one-hour drive away. This distance creates a considerable barrier to access HCV management access.

### Model-of-care

**Fig. 1:**
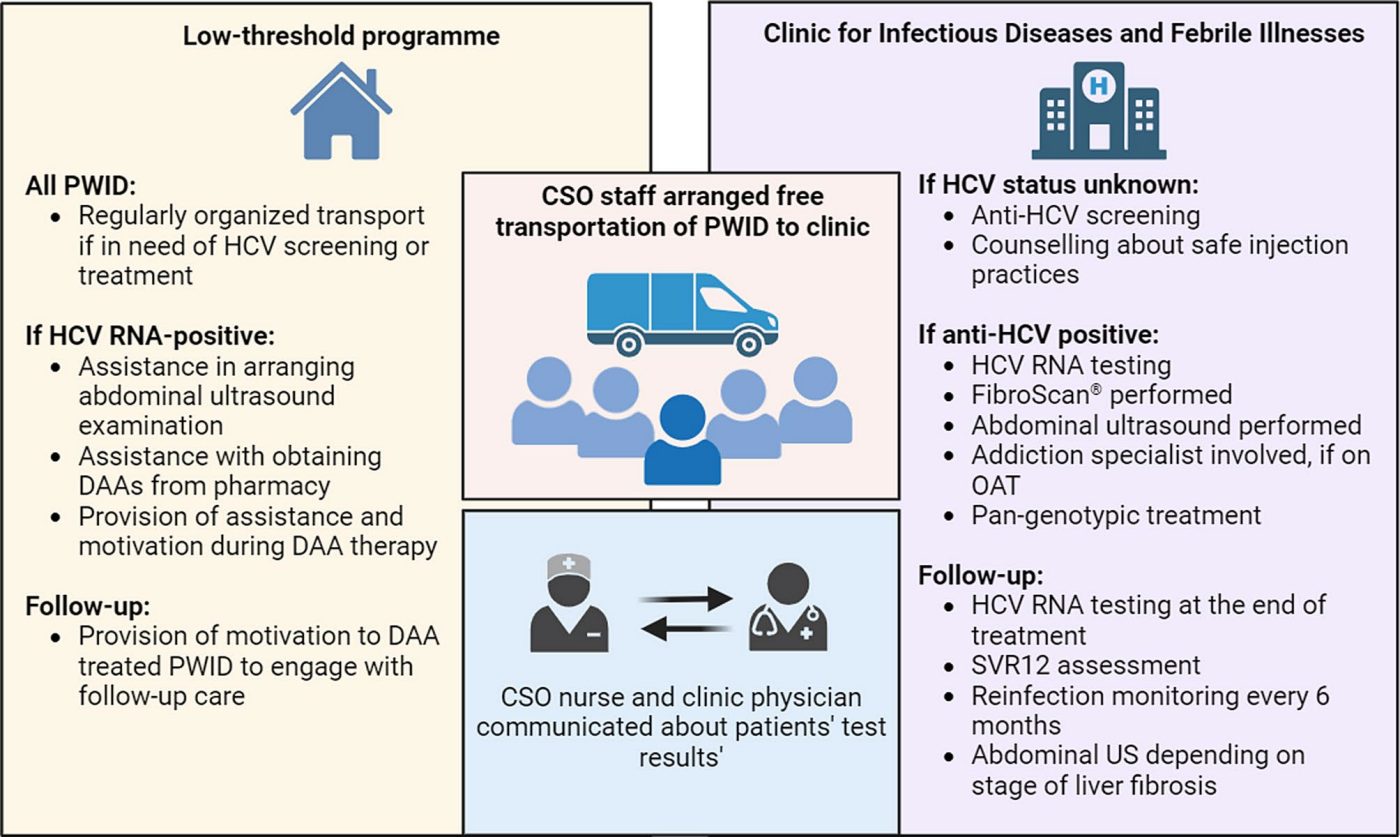
A model-of-care for the management of hepatitis C virus infection in people who inject drugs via the low-threshold programme. Created with BioRender.com. Abbreviations: CSO – civil society organisation, DAA – direct-acting antiviral, HCV – hepatitis C virus, OA T– opioid agonist treatment, PWID – people who inject drugs, SVR12 – sustained virological response 12 weeks after the end of treatment, US – ultrasound

The MoC consisted of regular, weekly transportation of all PWID, that were willing to receive HCV care from the LTP in Koper to the Clinic in Ljubljana, as arranged by the CSO. A dedicated viral hepatitis expert at the Clinic offered counselling and continuous HCV care, including anti-HCV screening, HCV RNA confirmatory testing, further diagnostics including transient elastography (FibroScan^®^), referral to liver ultrasound and other examinations, such as esophagogastroduodenoscopy in case of cirrhosis, introduction of pan-genotypic DAAs and follow-up (FU) during treatment, as well as FU after achievement of SVR12, in case of advanced liver disease. Regular HCV re-testing after SVR12 was routinely offered up to twice per year, and particularly in case of potential reinfection, with re-treatment being introduced as needed (Fig. 1). The staff from CSO gave out leaflets and information on hepatitis C treatment and provided information about hepatitis C in person in the day centre, on the field, and also by telephone. They provided transportation for PWID to the Clinic in Ljubljana, helped in referring PWID to various examinations outside of the Clinic, assisted patients with their DAA therapy, including directly overseeing treatment, and attempted to motivate those who were lost to FU (LFU) during or after DAA therapy, as well as communicated directly with the dedicated viral hepatitis expert at the Clinic, if needed.

### Patients

All PWID aged ≥ 18 years who were managed in the LTP at the CSO Svit Koper, anti-HCV positive and willing to use the MoC between January 2017 and December 2022 were included in the study. Previous HCV treatment was not an exclusion criterion. While anti-HCV screening was available as part of the MoC, some patients were screened at a CTIPDU or hospital and some were LFU during HCV care at the Clinic, prior to the study. Reinfection was defined as detection of HCV viraemia in PWID after completing DAA therapy and achieving SVR12.

### Statistical analysis

Baseline characteristics were reported as means with standard deviations for numerical variables and counts and percentages for categorical variables. Confidence intervals were calculated for the proportions using binomial exact calculation.

The time to reinfection represents the period between the date of first HCV RNA negative test after HCV treatment completion (SVR12) to the first positive HCV RNA re-test in patients, who were part of our MoC and achieved SVR12. The patients were tested up to twice per year and the date of the last test was recorded for all patients at risk. The maximum follow-up date for each patient was limited by the end of 2022 or death, whichever happened earlier. The data are interval censored – the exact time of reinfection is not given; we only know the reinfection had happened at some point since the last negative HCV RNA test to the first positive HCV RNA re-test. Both the interval censoring and the censoring due to the end of follow-up can be regarded as non-informative as we assume it is not related to reinfection risk.

For comparability with other studies, we have estimated the rate of a positive test taking into account censoring. As our main result, we have estimated the reinfection rate and the probability of reinfection in time using the methodology for interval-censored data [19]. We’ve checked the changing of the reinfection hazard in time and estimated the average reinfarction rate (assuming constant hazard of reinfection).

Analyses were performed using IBM SPSS version 24 (IBM, Armonk, NY, USA), while package icpack in statistical software R was used to analyse interval-censored data [20].

### Ethics

This study was approved by the Slovenian National Medical Ethics Committee (consent number 0120–622/2020/11) in July, 2020.

## Results

### Patients’ characteristics

During the observational period between 2017 and 2022, there was an average of 230 PWID (minimum 194 PWID, maximum 271 PWID) annually registered to the LTP Svit Koper, 191 (85.6%) males with the average age of 50.2 years. Out of them 49 met the inclusion criteria and were analysed in the study; their baseline characteristics are presented in Table 1.

**Table 1:** Baseline characteristics of all hepatitis C virus seropositive people who inject drugs and who were included in the hepatitis C virus model-of-care (*N* = 49)

Variables	anti-HCV positive (*N* = 49)
Mean age (years)	**38.67 (SD = 7.6)**
Male	**40** (81.6%)
Diagnosis of psychiatric illness	**6** (12.2%)
Excessive alcohol use	**11** (22.5%)
Reported recent injecting drug use	**26** (53.1%)
Previous hospitalisations	
- Bacterial infections linked to IDU	**10** (20.4%)
- Psychiatric hospital	**11** (22.5%)
OAT therapy	**42** (85.7%)
Psychotropic therapy	
Benzodiazepines	**28** (57.1%)
Sedative hypnotics	**15** (30.6%)
Antipsychotics	**17** (34.7%)
Antidepressants	**14** (28.6%)
Clinical picture of cirrhosis	**7** (14.3%)
Previously incarcerated	**16** (32.7%)
Experiencing homelessness	**14** (28.6%)
Death	**6** (12.2%)

*Abbreviations*: IDU – intravenous drug use, HCV – hepatitis C virus, OAT – opioid agonist treatment

Of the 49 anti-HCV positive PWID, 26 (53.1%) were first introduced into HCV care as part of the MoC. In 9/26 (34.6%), 10/26 (38.5%), and 7/26 (26.9%) of these, a positive anti-HCV test was diagnosed for the first time at the Clinic, CTIPDU, and while receiving medical care for other conditions, respectively.

**Fig. 2:**
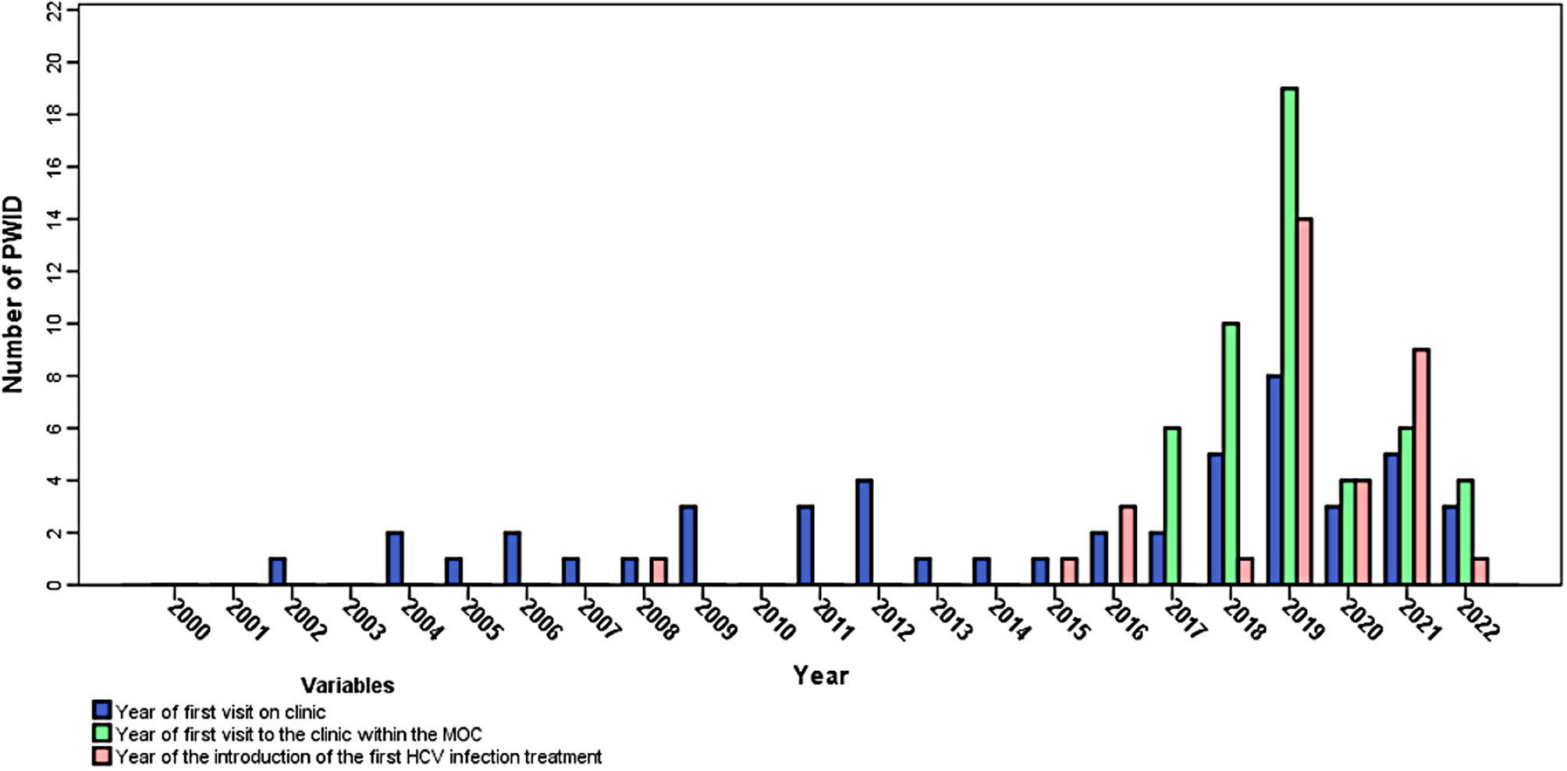
Timeline of the first visit to the Clinic and the first introduction to hepatitis C virus treatment among hepatitis C virus infected people who inject drugs included in the model-of-care (*N* = 42). Abbreviations: HCV – hepatitis C virus, MoC – model-of-care, PWID – people who inject drugs

A total of 20/26 (76.9%) of these participants tested positive for HCV RNA within the MoC. The remaining 23/49 (46.9%) anti-HCV positive PWID had already been introduced into some kind of HCV care prior to MoC engagement. Among them, 21/23 (91.3%) had been previously diagnosed as HCV RNA-positive, with 5/21 (23.8%) initiating HCV treatment and achieving SVR12; however, 22/23 (95.7%) had been LFU before the start of the study, and were then retrieved back into HCV care within the MoC, once it started to operate. A timeline including information on participants´ first visit to the Clinic and their first introduction to HCV treatment is presented in Fig. 2.

Of the 49 anti-HCV positive PWID, 8 (16.3%) had spontaneous HCV infection clearance, yet 1/8 (12.5%) presented with reinfection while under follow-up and was thus included among those who were HCV RNA-positive (Figure S1). In total 42/49 (83.7%) participants were HCV RNA-positive, 6/42 (14.3%) died (one due to decompensated cirrhosis, one due to advanced chronic kidney failure, and in the rest the cause of death was not apparently correlated to their HCV infection). The mean age among those who died was 49.1 years (SD = 4.8 years, minimum: 44 years, maximum: 57 years).

### Liver fibrosis evaluation

Of 42 HCV RNA-positive PWID, 37 (90.2%) had a liver fibrosis evaluation by transient elastography (FibroScan^®^). Overall, 13/37 (35.1%) had a stage of fibrosis > F2 according to the METAVIR scoring system. Clinical signs of cirrhosis were present in 6/42 (14.3%), oesophageal varices in 2/42 (4.8%) and ascites in 2/42 (4.8%) HCV RNA-positive PWID.

### Treatment of hepatitis C

A total of 36/42 (85.7%) HCV RNA-positive PWID started DAA therapy, with an average age of 42.72 (SD = 5.71) years at treatment initiation, while 3/42 (7.1%) were LFU before starting treatment, 2/42 (4.7%) were in the process of starting DAAs, and 1/42 (2.4%) died prior HCV treatment initiation. A study tree is presented in Figure S1 and the specific therapy for treating hepatitis C that the participants received is shown in Table S1.

A total of 5/36 (13.9%) participants received HCV treatment prior to MoC initiation, while the other 31/36 (86.1%) participants received their first HCV treatment within the study. Of these, 28/31 (90.3%) participants completed treatment, 1/31 (3.2%) died during the treatment due to reasons not related to their HCV infection and 2/31 (6.3%) were currently receiving treatment. Of those who completed treatment, 23/28 (82.1%) achieved SVR12 and 5/28 (17.9%) presented with ETR but were LFU after treatment completion. Of all the treated participants, 33/36 (91.7%) completed treatment, of whom 28/33 (84.9%) achieved SVR12, and 5/33 (15.2%) presented with ETR but were LFU after completing treatment.

**Fig. 3:**
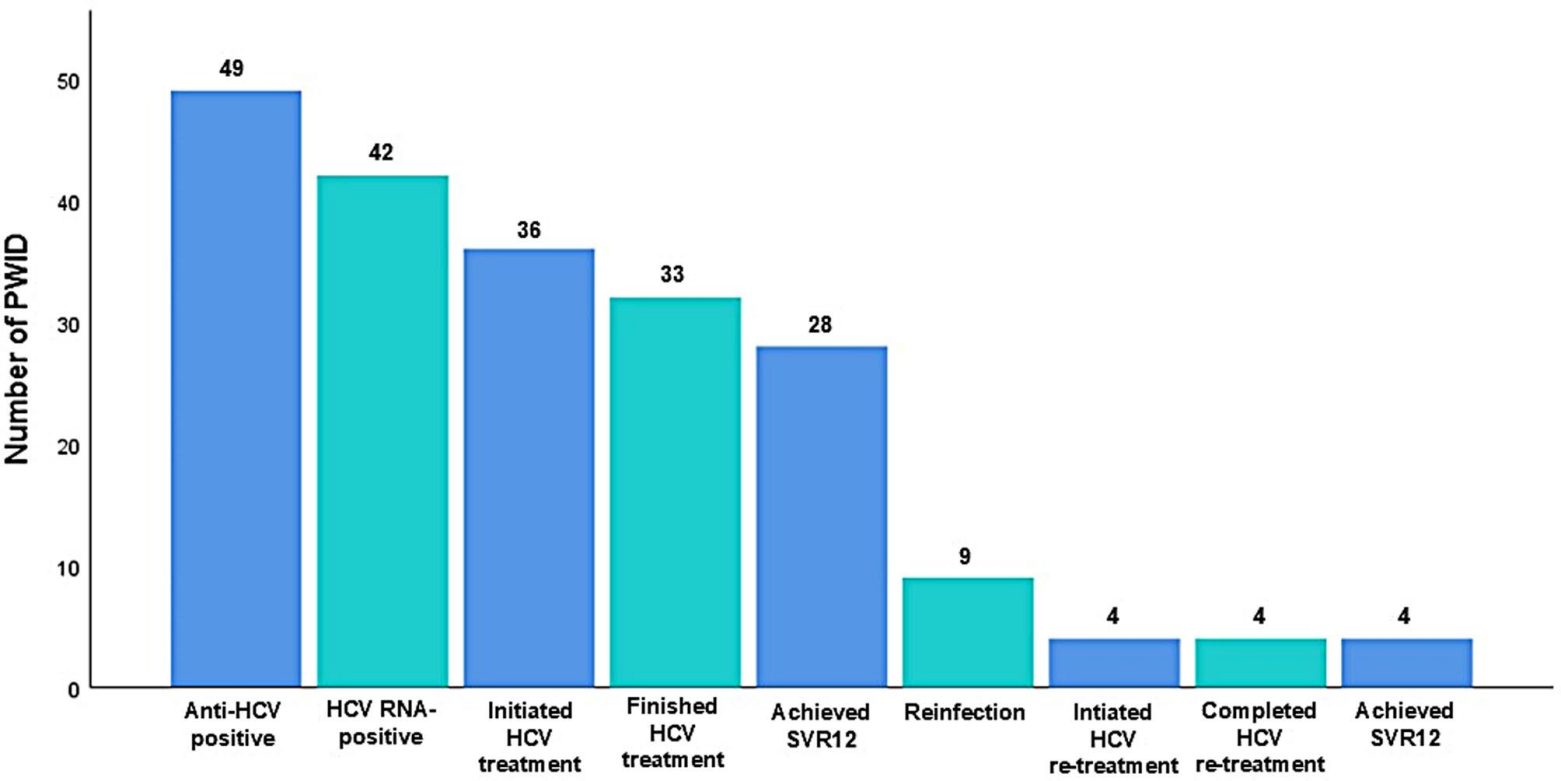
The cascade of hepatitis C care within the model-of-care for people who inject drugs (*N* = 49). Abbreviations: HCV – hepatitis C virus, PWID – people who inject drugs, SVR12 – sustained virological response 12 weeks after the end of treatment

The average number of MoC visits before DAA initiation per one participant was 6.0 (SD = 4.58, minimum: 1, maximum: 23), with 18/36 (50%) having their first visit at the Clinic (Fig. 2). The HCV cascade-of-care within the MoC is shown in Fig. 3.

### Reinfection

Of 33 PWID who achieved ETR, 28 (84.9%) patients achieved SVR12 during observation period and were thus included in the analysis of reinfection risk. 22 of these patients were not reinfected at the time of their last testing within the study period, and in 6 reinfection was recorded during the follow-up period. The average FU period was 21.0 months, ranging from 2.4 to 47.9 months. The rate of a HCV RNA-positive re-test equals 12.2 per 100 person-years (95%CI [7.7–16.7]); the rate of reinfection is estimated to equal 13.3 per 100 person-years (95%CI [6.0,29.7]).

**Fig. 4:**
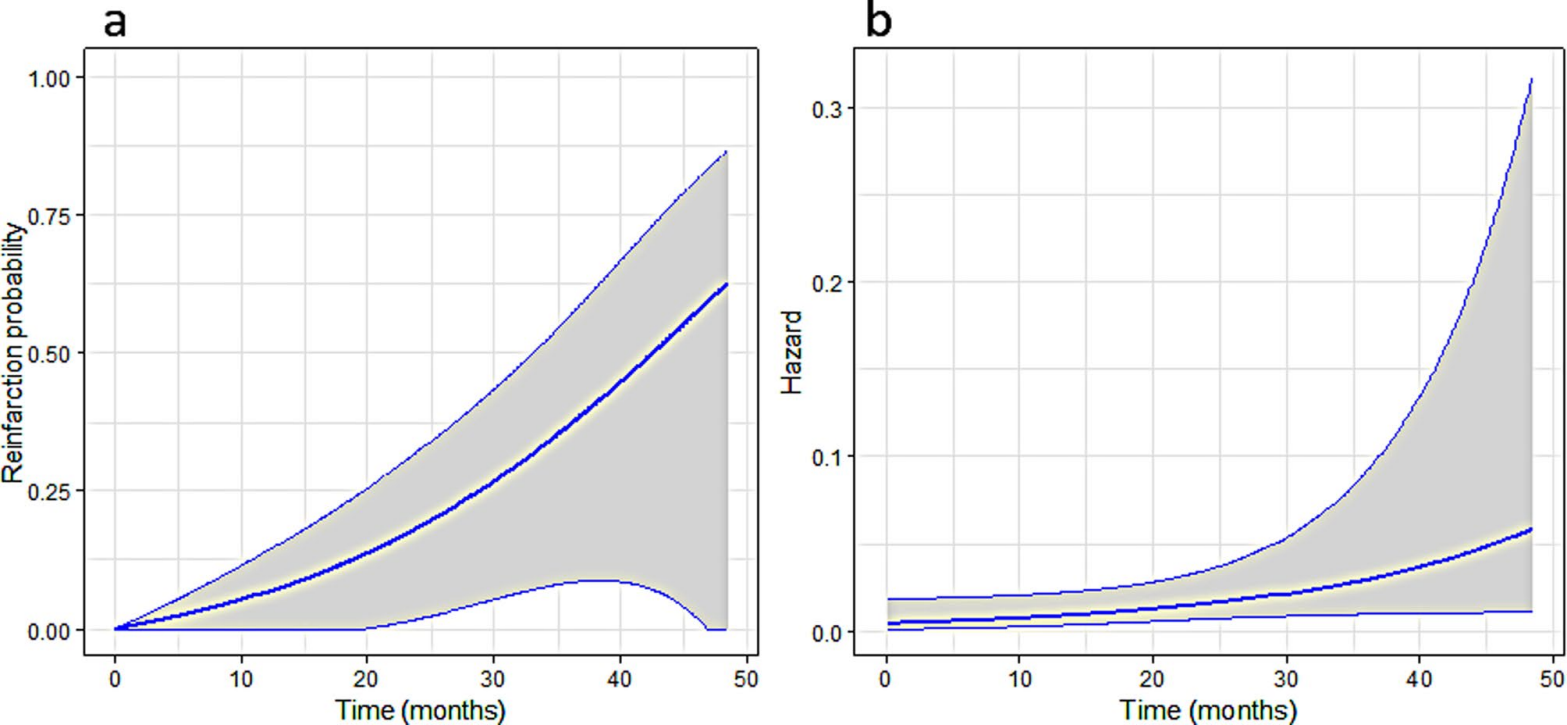
The estimated probability of reinfection (**a**) and the estimated hazard of reinfection in time (**b**) for people, who inject drugs and who achieved sustained virological response 12 weeks post treatment as part of a model-of-care (*N* = 28)

The estimated probability of reinfection is given in Fig. 4a. The estimated reinfection probability exceeds 0.5 at 4 years, but the confidence intervals are quite wide. Figure 4b shows the estimated hazard of reinfection in time, we can see that there is no indication it would be decreasing in time, in fact, it is increasing on our sample.

Among those reinfected, 2/6 had completed HCV re-treatment, 3/6 were in the process of re-treatment initiation, and one died before HCV re-treatment initiation from non-HCV related reasons (Figure S1).

Their mean age at treatment for their first infection was 43 years (minimum: 39 years, maximum: 53 years) and the mean time to positive HCV RNA test was 29 months (minimum: 13 months, maximum: 48 months).

In addition to managing those six reinfected individuals that achieved HCV cure within the MoC, two PWID were managed due to reinfection after being treated for HCV elsewhere and another experienced spontaneous HCV clearance during the MoC presented with reinfection but died prior to initiation of DAA therapy, so altogether 9/49 (18.4%) PWID had HCV reinfection in observed cohort. Their characteristics are summed up in Table 2 and received HCV therapy is summed up in Table S1. The characteristics of all PWID with reinfection are summed up in Table 2.

**Table 2:**
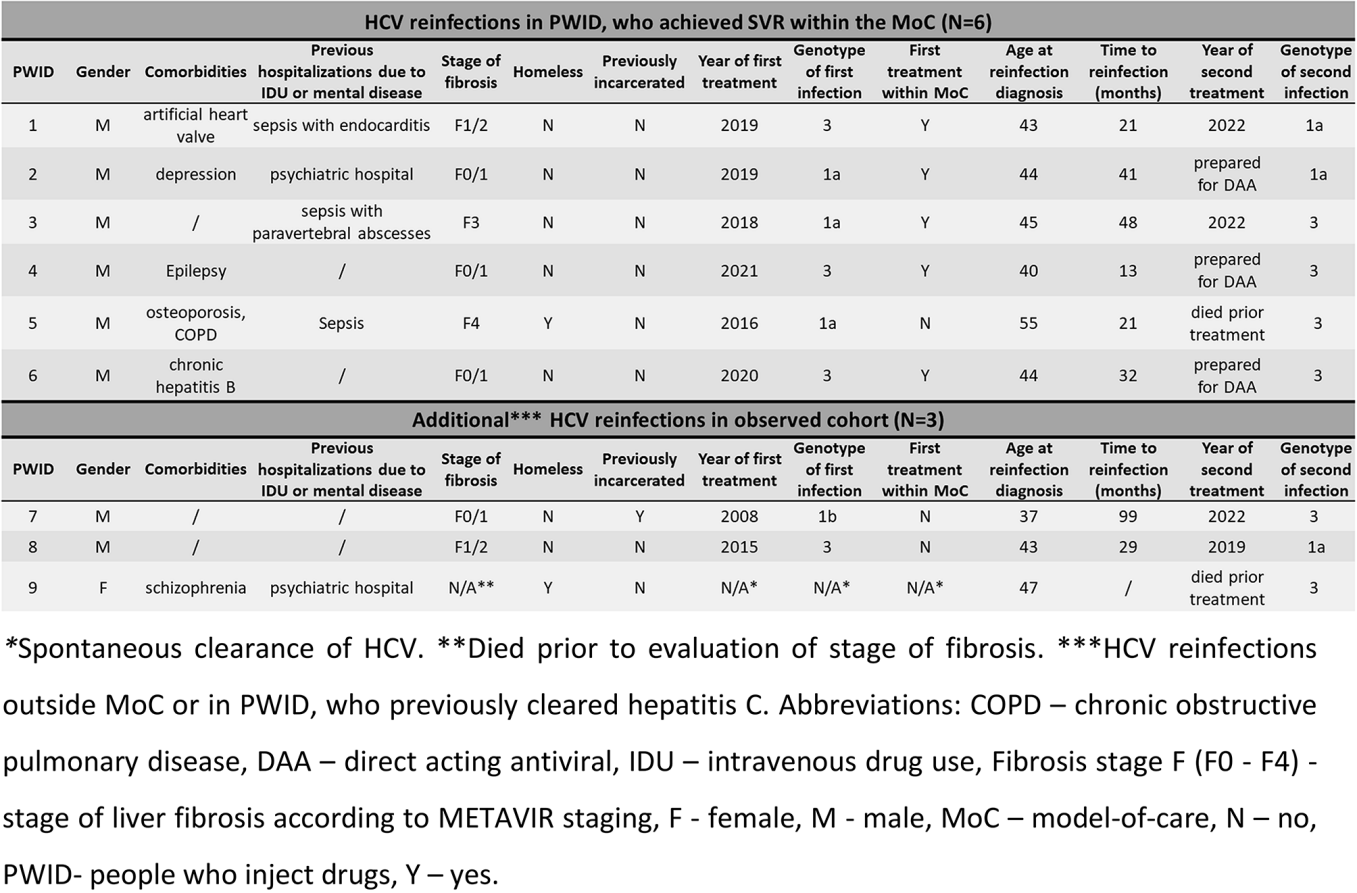
Characteristics of people who inject drugs with a hepatitis C virus reinfection, managed within the model-of-care (*N* = 9)

## Discussion

This study examined the effectiveness of a novel model of HCV care in Slovenia in a high-risk cohort for HCV infection within an LTP.

Slovenia has a relatively low estimated prevalence of chronic HCV infection (0.07%), and according to the latest modelling study, in 2019, approximately 1,078 individuals living with chronic hepatitis C still needed HCV treatment [11]. Among those, 407 were estimated to be recent PWID and 441 former PWID with the estimated HCV RNA prevalences of 5.2% and 13.5%, respectively [11]. Since currently in Slovenia the majority of those in need for HCV treatment belongs to this vulnerable and marginalised population, tailored interventions to address its specific needs are especially important on the way to achieve HCV micro-elimination.

Our MoC was founded based on the results of the two national studies performed in 2017 in LTPs across the country, presenting anti-HCV seroprevalence of 38% among PWID that were using their services [21, 22]. Even though many of them had been routinely tested and found HCV RNA-positive before the introduction of MoC at the local CPTIDUs, where they were receiving OAT, or elsewhere, they had declined to be linked to HCV care and get cured, however their HCV status in the LTP registers remained unknown.

Multiple studies have shown that decentralised healthcare services with test-and-treat approach in community settings can improve the HCV cascade-of-care and result in high uptake of treatment with DAAs among PWID [23–26]. However, in our MoC HCV therapy was prescribed within the healthcare system, while specific interventions (e.g. organised transportation and physician, nurse, and social worker support) were implemented to improve the HCV cascade-of-care, by simplifying access to disease management, and improving knowledge regarding HCV transmission among this high-risk population. At the same time, efforts were made to maintain a high standard of care for the treatment of liver disease and other comorbidities, with a focus on follow-up and screening for reinfection after HCV cure [27, 28]. The latter was difficult to achieve as socioeconomic and mental health problems were especially pronounced in this highly marginalised and vulnerable cohort of patients, resembling observations made in other studies conducted among similar groups [29, 30].

Despite the social and economic challenges of the study cohort and health-care setting, the MoC notably improved HCV management and linkage-to-care within this population. While prior to the introduction of the MoC a half of HCV RNA-positive PWID in our observed cohort had at least one clinic visit, only a quarter received HCV treatment and the rest were LFU. Within the MoC, though, 86% started treatment and only 7% were LFU before treatment initiation. Since a known HCV-positivity was not a prerequisite for participation in the organised transport and physician visits, even PWID who had previously cleared HCV attended regular visits for reinfection screening, and one reinfection was detected during such a FU. Although the MoC successfully improved the HCV cascade-of-care in this cohort compared to LTP baseline prior to MoC (Fig. 2), SVR rate was slightly lower yet still comparable to rates reported in similar studies published by Valencia et al., Schwartz et al., and Midgard et al. (90.7% vs. 90.7% vs. 94.7% vs. ours 84.9%, respectively) [30–32], and there is a need for future outreach and treatment of those PWID who were LFU.

While HCV reinfections were rarely detected in the pre-DAA era [33], the issue has recently come to the forefront as the possibility of HCV reinfection remains one of the major obstacles on the path to complete HCV elimination. While some studies have shown that HCV reinfection rates have been low, even in the DAA era [34, 35] and among PWID (reinfection rate of 6.2 per 100 - PY in a meta-analysis by Hajarizadeh et al.) [36], others have reported higher reinfection rates of more than 20 per 100 - PY among currently injecting PWID [37, 38] and incarcerated people [37, 39].

In our study, we took in the account, that the time to the positive HCV RNA re-test was different than the time of actual reinfection, thus separately calculating the reinfection rate, which was 13.3 per 100 - PY, while the rate of positive HCV RNA re-test equaled 12.2 per 100 – PY, which is higher than the rates reported by Valencia et al. (9.8 per 100 - PY), Midgard et al. (3.74 per 100 - PY) and Litwin et al. (11.4 per 100 - PY) [30, 32, 40], but lower than the highest reported reinfection rates among current PWID in studies by Hibbert et al. (22.55 per 100 - PY) and Schulkind et al. (and 21.5 per 100 - PY) and Cunningham et al. (17.9 per 100 - PY) [37, 38, 41]. Additionally, the mean time to reinfection in our study was 29 months, which was longer than 7.2 months reported by Valencia et al. and 28.5 weeks observed by Midgard et al. [30, 32]. The longer mean time to reinfection observed in our study may be attributed to a longer observation period of six years, with a maximum reinfection follow-up of 48 months and the longer average FU period of 1.75 years (interquartile range (IQR) 0.8–2.7 years). In comparison, Valencia et al. reported an observation period of 34 months, and Midgard et al. reported a follow-up period of 0.5 years (IQR 0.23–1.40 years).

Additionally, the availability of harm reduction services and increased awareness of HCV transmission risks among participants also may have contributed to the delayed onset of HCV reinfection. Moreover, even though most studies reported a decrease in reinfection incidence over time after SVR [36, 40], our estimated reinfection probability exceeded 0.5 at 4 years, with the hazard of reinfection increasing over time.

In addition, previous studies have shown that PWID receiving OAT were less likely to be reinfected [33]. However, in our cohort, all PWID with reinfection were receiving OAT and surprisingly 86% of all our anti-HCV positive PWID were also receiving OAT within CPTIDUs, which is similar to 87% in a study from Valencia et al. [30], but higher than in most published LTP studies [32, 38, 42]. Since OAT is a part of a high-threshold programme for PWID, it is important to be vigilant in screening for injection relapse as well as HCV RNA, and to promptly recognise an increased risk for infection and reinfection with HCV and other bacterial infections associated with IDU. In the DAA era, it is extremely important to monitor all PWID on OAT after successful HCV treatment, not only for liver disease advancement, but also for the possibility of HCV reinfection.

Advanced liver disease was also prominent in the studied cohort as 14% of our anti-HCV positive PWID had clinical signs of cirrhosis and 36% had a METAVIR stage > F2 on FibroScan^®^. Compared to the studies by Midgard et al. [32] and Kåberg et al. [43], the proportion of patients with advanced liver fibrosis in our study was higher (29.1% vs. 15% vs. 36%, respectively) but the proportion of excessive alcohol use was lower (38% vs. 34% vs. 23%, respectively), suggesting that HCV infection is an important cause of advanced liver fibrosis in the studied cohort. Moreover, a national study performed in Slovenia in 2018 on liver fibrosis in HCV-infected PWID attending LTPs showed 26.1% of late presenters [22], while in our study, the proportion in LTP Svit Koper was even higher, probably due to socioeconomic challenges of the observed cohort and the absence of linkage-to-care before the initiation of MoC.

The main advantage of our study is that the clinical goal of improving the cascade of HCV care and particulary linkage-to-care was achieved for this vulnerable population using a new MoC with improved rates of treatment initiation and completion. The possibility of HCV reinfection was monitored among participants not only after being cured with DAAs, but also after spontaneous clearance of HCV, with one reinfection being detected in the latter. Despite the relatively small number of participants in the MoC, the collected data allowed us to calculate the reinfection rate, the rate of positive HCV RNA re-test, and the hazard of HCV reinfection with time. Although the results of our study cannot be generalised, such a MoC could serve as an example of a successful collaboration between a LTP and a hepatitis treatment centre, in a country where treatment of HCV is done with no limitations within the national and regional healthcare systems. Furthermore, our study highlights that in such a population, completing HCV treatment and achieving SVR12 do not present the endpoints of a cascade-of-care as consistent follow-up, continuous screening for HCV reinfection and re-treatment are essential to achieve and sustain HCV elimination as in our sample hazard of reinfection with time even increased.

Our study and MoC faced some limitations and barriers to more effective HCV care, the first one being our limited knowledge on HCV burden among the users of LTP Svit Koper, since no on-site HCV testing was available. While annually there were on the average 230 PWID anonymously registered in this LTP, and the estimates of HCV prevalence in LTP population and in recent PWID in Slovenia were considered from previous national studies [11, 21], the heterogeneity of the LTP users and the lack of on-site testing limited our ability to accurately assess the true size of our target population, which included anti-HCV positive PWID within LTP Koper Svit. As a result, the analysed MoC cohort offers a limited insight into our target population and further studies are needed. Moreover, HCV testing that was conducted off-site, e.g. at the Clinic in Ljubljana, local CTIPDUs, or elsewhere, posed additional logistical challenges that likely reduced participation in the MoC screening, and severely impacted our first step of a cascade of care.

The second limitation was the inability to determine the uptake rate of our MoC in target population of LTP, as due to anonymous nature of LTP registry, there was no available data on HCV status of LTP users, even if there had previously been tested off-site. Our cohort most likely reflects those, who were more motivated to engage with HCV treatment, which may not accurately represent the broader population of LTP Svit Koper users. Taken together, more localized services, that include on-site HCV testing and HCV treatment in community settings are needed, to avoid barriers to linkage-to-care and enhance access to HCV therapy and cure.

Third barrier was the loss to follow-up. Despite high engagement from the doctor, nurse and social workers involved in MoC there was still 7% of PWID who were LFU before HCV treatment and 15% after ETR. Although transport to the Clinic in Ljubljana was required for treatment, visits were available weekly for PWID interested in accessing HCV care. However, the logistical burden of traveling may had still deterred some participants, further emphasizing the need for more localized services or even a mobile unit implementing a test-and-treat service. This approach would not only improve accessibility of HCV care and enable prompt treatment initiation, but also enhance engagement with PWID to reduce loss to follow-up.

Despite the small cohort size, the major contribution of our study is that, to our knowledge, this is the first study to estimate the hazard of HCV reinfection with time, revealing an increasing trend in our sample, in contrast to previously reported studies. This highlights that, despite the availability of harm reduction measures such as needle and syringe exchange programs and awareness of HCV transmission, additional interventions are necessary. For example, introducing safe injecting room could further support ongoing efforts to reduce HCV transmission within this population.

## Conclusions

In conclusion, the study results demonstrate an improved HCV cascade-of-care in a difficult-to-treat and vulnerable population in a real-world setting, which was achieved through a newly established MoC. While the size of the total targeted population in need for DAAs could not be determined, the outcomes of DAA treatment in those included were favourable. Importantly, our longer observational follow-up period post cure revealed higher rates of reinfection compared to previous studies and in our sample hazard of reinfection with time even increased, despite the availability of harm reduction services. These findings highlight that in the future, MoC should be improved by on-site HCV testing and treatment within community. Since reinfections obviously present an important obstacle on the way to HCV elimination, achieving micro-elimination in this cohort will require ongoing monitoring, not only to address liver disease progression but also to manage the potential for reinfections among PWID, both after DAA therapy and in those who have cleared HCV spontaneously.

## Electronic supplementary material

Below is the link to the electronic supplementary material.

Supplementary Material 1

## Data Availability

Data is provided within the manuscript or supplementary information files.

## Abbreviations

Anti-HCV: HCV antibody
Anti-HBc: Antibody against hepatitis B virus core antigen
CKD: Chronic kidney disease
COPD: Chronic obstructive pulmonary disease
CPTIDU: Centre for Prevention and Treatment of Illicit Drug Use
CSO: Civil society organisation
CVD: Cardiovascular diseases
DAAs: Direct-acting antivirals
ETR: End of treatment response
Fibrosis stage F: Stage of liver fibrosis according to METAVIR staging
FU: Follow-up
HBV: Hepatitis B virus
HCV: Hepatitis C virus
IDU: Intravenous drug use
LFU: Lost to follow-up
LTP: Low-threshold programme
MoC: Model-of-care
OAT: Opioid agonist treatment
PWID: People, who inject drugs
PY: Person-year
SVR12: Sustained virological response 12 weeks after end of treatment
