# Supplementary material for: Addressing viral hepatitis C reinfections in a low-threshold programme for people who inject drugs in Slovenia
Source: Harm Reduct J. 2025 Feb 13;22:16. doi: 10.1186/s12954-025-01164-5 (PMC11823079; doi:10.1186/s12954-025-01164-5)
Supplement: Supplementary file 1 — Supplementary Material 1 [file 12954_2025_1164_MOESM1_ESM.docx]

**SUPPLEMENTARY MATERIAL**

**Addressing viral hepatitis C reinfections in a low-threshold programme for people who inject drugs in Slovenia**

Jasna Černoša^1,7^, Jelka Meglič Volkar^1^, Mario Poljak^2^, Maja Pohar Perme^3^, Jeffrey Victor Lazarus^4,5,6^, and Mojca Matičič^1,7^

^1^ Clinic for Infectious Diseases and Febrile Illnesses, University Medical Centre Ljubljana, Ljubljana, Slovenia

^2^ Institute of Microbiology and Immunology, Faculty of Medicine, University of Ljubljana, Ljubljana, Slovenia

^3^Department of Biostatistics and Medical Informatics, Faculty of Medicine, University of Ljubljana, Ljubljana, Slovenia

^4^ Barcelona Institute for Global Health (ISGlobal), Hospital Clínic, University of Barcelona, Barcelona, Spain

^5^ City University of New York Graduate School of Public Health and Health Policy (CUNY SPH), New York, NY, USA

^6^ Faculty of Medicine and Health Sciences, University of Barcelona, Barcelona, Spain

^7^ Faculty of Medicine, University of Ljubljana, Ljubljana, Slovenia

**Corresponding author:** Jasna Černoša ([jasna.cernosa@kclj.si](mailto:jasna.cernosa@kclj.si))


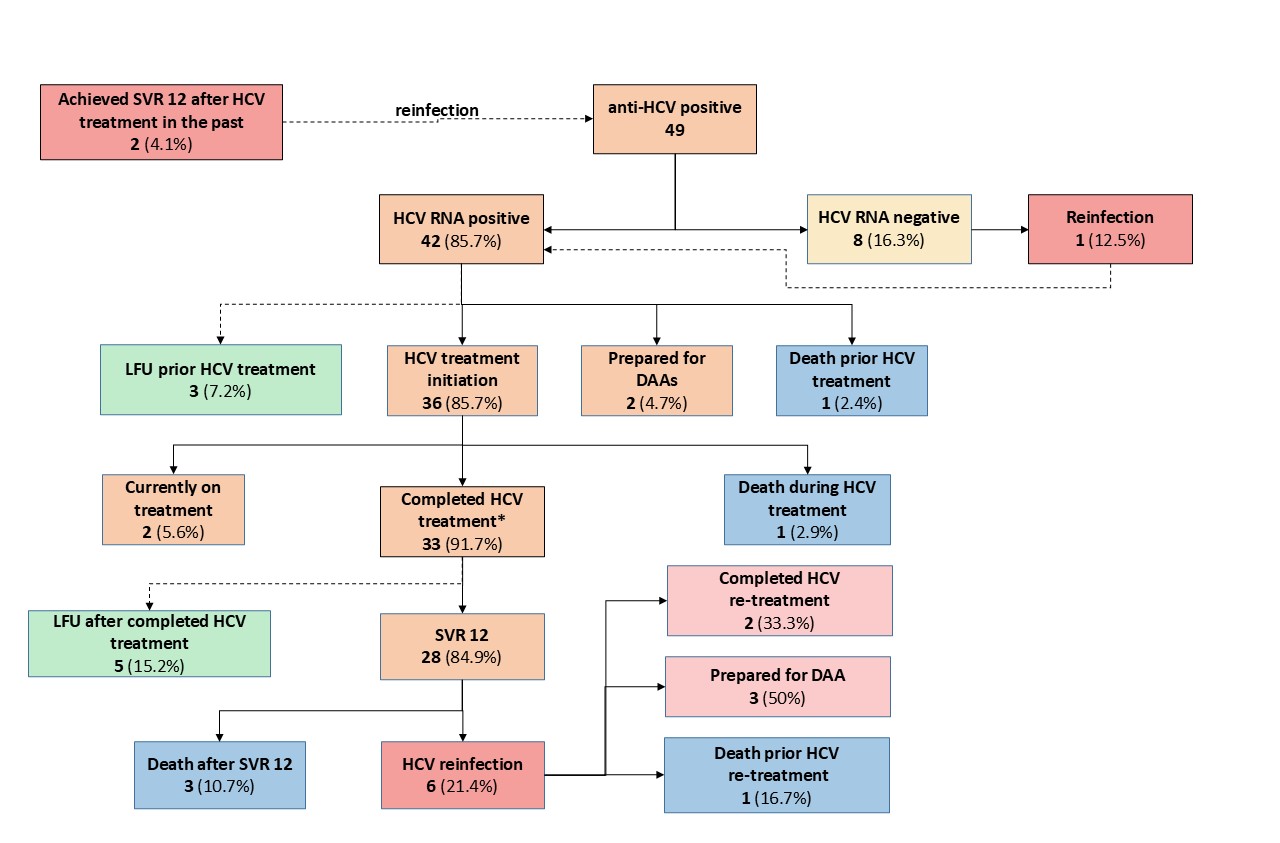


**Figure S1**: The study tree of all people who inject drugs managed within the model-of-care for hepatitis C.

*One person ended the treatment early but achieved sustained virological response 12 weeks after treatment and is included among those who completed HCV treatment. **Abbreviations:** DAAs – direct-acting antivirals, HCV – hepatitis C virus, LFU – lost to follow-up, SVR12 –sustained virological response 12 weeks after end the of treatment.

**Table S1:** Specific therapy for treating hepatitis C, received by participants, who initiated treatment

| Therapy of HCV infection | HCV RNA-positive, who initiated treatment (N=36) |
| --- | --- |
| Interferon-based regimen* | **3** (8.3%) |
| First-generation DAAs* | **2** (5.6%) |
| Sofosbuvir-velpatasvir | **14** (38.9%) |
| Glecaprevir/pibrentasvir | **14** (38.9%) |
| Ledipasvir/sofosbuvir | **1** (2.8%) |
| Elbasvir/grazoprevir | **2** (5.6%) |
| Therapy of HCV re-infection | **Reinfected with HCV, who initiated**  **re-treatment (N=4)** |
| Sofosbuvir/ledipasvir | **1** (25%) |
| Sofosbuvir/velpatasvir | **3** (75%) |

*Received the treatment before the initiation of a model-of-care

**Abbreviations:** DAAs – direct-acting antivirals, HCV – hepatitis C virus
